# Phrenic Nerve Block for Intractable Hiccups: A Case Series

**DOI:** 10.7759/cureus.96975

**Published:** 2025-11-16

**Authors:** Arun Kalava, Seema Parikh, Parker Nguyen, Caitlyn Ko, Kesava K Mandalaneni

**Affiliations:** 1 Department of Anesthesiology, University of Central Florida College of Medicine, Tampa, USA; 2 Department of Rehabilitation and Regenerative Medicine, New York-Presbyterian Hospital-Columbia and Cornell, New York, USA; 3 William Beaumont School of Medicine, Oakland University, Rochester, USA

**Keywords:** hiccups management, interventional pain therapy, intractable hiccups, nerve block technique, phrenic nerve block, post-surgical complications, ultrasound-guided procedures

## Abstract

Phrenic nerve block (PhNB) has emerged as a therapeutic option for intractable hiccups, defined as those persisting for more than 30 days. While previous case reports have shown success with this procedure, particularly in post-surgical cases, its efficacy across different patient populations remains uncertain. We present seven cases of intractable hiccups treated with PhNB that demonstrated limited or no therapeutic benefit. The cases include both idiopathic and post-procedural etiologies, with hiccups lasting from four months to 13 years. Patients underwent ultrasound-guided PhNBs using local anesthetic and perineural dexamethasone, with some patients receiving bilateral treatments. Despite initial relief in some cases, the therapeutic effect was either temporary or absent, with one patient experiencing severe post-procedure exacerbation of symptoms. Various alternative treatments were attempted, including peripheral nerve stimulation devices, with varying success. This case series highlights the variable response to PhNB in treating intractable hiccups and emphasizes the need for careful patient selection. It also underscores the importance of developing standardized protocols for anesthetic dosing and procedural techniques, while considering alternative therapeutic approaches for this challenging condition. These cases contribute to the growing body of literature on interventional treatments for intractable hiccups and highlight the complexities in managing this condition.

## Introduction

There are many indications for phrenic nerve block (PhNB), including intractable hiccups, diaphragmatic paralysis, and thoracic outlet syndrome [1,2]. Intractable hiccups, in particular, are not uncommon in clinical settings and are thought to be caused by an interruption to the hiccup reflex arc, which causes diaphragmatic contractions. Patients often report using conservative measures like drinking carbonated beverages, traditional acupuncture, or physical maneuvers like breath-holding or induction of emesis in an attempt to manage their symptoms [3].

The definition of "persistent hiccups" is hiccups that persist for more than 48 hours or recur frequently and may be due to various causes, such as post-surgical complications, neurological conditions, or gastrointestinal issues. "Intractable hiccups," in contrast, persist for more than 30 days and can lead to significant morbidity, including increasing the need for surgical revisions and prolonged hospital stays [4]. Long-term hiccups can give rise to various complications, including dehydration, weight loss, fatigue, insomnia, and depression [5]. PhNB has been shown to be an effective treatment for postoperative intractable hiccups in certain patient populations, often providing rapid relief [3]. However, some patients may be resistant to PhNB, or the effect may be short-lived. In this case series, we present seven patients with intractable hiccups who underwent PhNB and had variable responses.

Five of the seven cases had idiopathic, intractable hiccups that were not resolved with conservative measures. The remaining two cases had post-procedural intractable hiccups from an atrial ablation and cortisone injections, respectively [6]. The following cases will discuss challenges in identifying suitable candidates for PhNB, limitations of the procedure, potential adverse effects of the procedure, and overall procedural outcomes.

## Materials and methods

This study is an observational, descriptive case series based on a retrospective review of seven patients who presented with intractable hiccups and underwent ultrasound-guided PhNB at an outpatient interventional pain clinic. The primary objective was to describe patient characteristics, procedural details, and variability in clinical response, rather than to evaluate efficacy in a controlled manner. Intractable hiccups were defined as episodes persisting for more than 30 consecutive days despite standard conservative management. All patients included in this series had previously attempted at least one form of therapy, pharmacologic or non-pharmacologic, without adequate relief. Cases were identified by reviewing clinic records, and pertinent clinical data were extracted from electronic medical records. Collected variables included patient demographics, medical and surgical history, duration and presumed etiology of hiccups, prior interventions, PhNB procedural parameters, and post-procedural outcomes. Prior diagnostic and imaging studies referenced in patient histories (e.g., colonoscopy, CT, endoscopy) were performed at outside institutions and are therefore unavailable for direct review.

All PhNBs were performed by a board-certified anesthesiology physician with expertise in ultrasound-guided regional anesthesia. Patients were placed in the supine position with the head turned contralateral to the side of the planned procedure. The phrenic nerve was identified under ultrasound guidance as it coursed anterior to the anterior scalene muscle, typically between the C3 and C6 levels, in close proximity to the carotid sheath and brachial plexus. A 22-gauge echogenic or stimulating needle was advanced under real-time ultrasound visualization, and in select cases, a nerve stimulator was also employed to elicit diaphragmatic contraction for confirmation of needle placement.

Following correct positioning, local anesthetic was injected adjacent to the nerve, most commonly ropivacaine (Case 1 utilized 0.33%; all other cases utilized 0.25%) or lidocaine (1%), combined with perineural dexamethasone in doses ranging from 4 to 8 mg. Injection volumes varied from 3 to 4 mL depending on the patient's anatomy and the side of the procedure. PhNBs were performed either unilaterally or bilaterally, depending on the persistence or recurrence of symptoms.

Patients were monitored during and immediately following the procedure for acute complications, such as respiratory compromise, hemodynamic instability, or neurological changes. Clinical outcomes were assessed based on patient-reported symptom relief immediately after the procedure, during follow-up visits within one week, and at subsequent visits when available. The outcomes of interest included change in hiccup frequency and severity, duration of symptom relief, and associated impacts on sleep, appetite, mood, and quality of life. Adverse events and procedural complications were also recorded. Given the small number of cases, no statistical analysis was performed, and results are presented descriptively in the individual case narratives.

## Results

Case 1

A 74-year-old African American male and retired physician, with a history of hypertension and prior hiatal hernia surgery six years ago, presented to the clinic with a 13-year history of intractable hiccups. He described a prodromal sensation of warmth and sweating preceding each episode, often accompanied by a small amount of clear sputum. In the days before his visit, he experienced a noticeable increase in hiccup frequency, associated with chest pain. The patient denied experiencing dysphagia, odynophagia, or weight loss.

The patient experienced temporary relief from hiccups by drinking carbonated water, which provided 10-15 minutes of respite. Notably, he had been admitted to the ICU five weeks earlier for hyponatremic seizures in the setting of excessive liquid intake. During that hospitalization, both upper and lower GI endoscopies showed no abnormalities. Previous treatments included acupuncture, which was ineffective.

At the time of presentation, the patient was taking promethazine with codeine syrup, risperidone, and famotidine for unrelated symptoms as part of his pre-existing medication history. On physical examination, he appeared to be in mild to moderate distress, actively experiencing hiccups and having concomitant secretions.

A right PhNB was performed using 3 mL of ropivacaine 0.33% and 4 mg of perineural dexamethasone under ultrasound guidance. The procedure was completed without complications. On follow-up, the patient reported feeling "good" until around 8 p.m. (four hours post-procedure, after administration at 4 p.m.) on the day of the procedure, when the relief abruptly stopped. In response, the treatment plan was adjusted to either target the left phrenic nerve or perform a stellate ganglion block. This patient failed to follow up post-procedure.

Case 2

A 54-year-old Caucasian male engineer with a history of asthma presented with chronic hiccups that had persisted for 12 years and had worsened significantly over the last two years following a COVID-19 infection. The condition initially manifested after an atrial ablation procedure in 2011, possibly due to damage to the right phrenic nerve. The patient experienced extreme and prolonged episodes of hiccups lasting for hours, often accompanied by feelings of breathlessness and a fear of suffocation. Notably, eating was the only intervention that provided relief, and alcohol was identified as a trigger for the hiccups. The patient's medication regimen included baclofen and pantoprazole for symptom relief.

The patient's diagnostic workup included chest fluoroscopy, which demonstrated normal diaphragmatic motion despite elevation of the right hemidiaphragm, indicating no evidence of paralysis or paresis. The cardiac silhouette and mediastinal contour were normal. A chest CT showed tiny pulmonary nodules (4 mm in the right upper lobe and 3 mm in the right lower lobe), basilar scarring, and a dilated ascending aorta measuring 4.1 cm. These findings were consistent with partial rather than complete right phrenic nerve dysfunction, aligning with the clinical suspicion of a residual, nonparalytic injury from prior ablation.

An attempt was made to perform a right PhNB as a prerequisite for qualifying for phrenic nerve surgery. The procedure was done under ultrasound and nerve stimulator guidance, using a 22-gauge stimulating needle (Pajunk Medical Systems, Alpharetta, GA, USA). However, due to anatomical variance, the phrenic nerve could not be visualized or stimulated during the ultrasound-guided procedure attempts at both the C-6 and C-4 levels, anterior to the anterior scalene muscle, and hence, the procedure was terminated. The patient planned to consult with their surgeon regarding the next steps.

Case 3

A 67-year-old Caucasian male, currently retired, with a history of hypertension and multiple joint replacements (bilateral knee, bilateral hip, and left shoulder), presented with chronic hiccups that had persisted for 2.5 years. The condition initially manifested following corticosteroid injections to his left shoulder but progressively worsened over time. He experienced four to five episodes daily, ranging from five minutes to several hours in duration and primarily triggered by eating. The hiccups could be temporarily relieved by drinking cold water or lying on his left side.

The patient's symptoms were managed with a medication regimen including baclofen and chlorpromazine, which had helped decrease the frequency of episodes, and gabapentin, which provided approximately 50% pain relief. Notably, the patient had no chest discomfort, dysphagia, or weight loss.

A series of interventional treatments was attempted. First, a left PhNB was performed. During follow-up two days later, the patient reported a 20% reduction in the severity of his hiccups after eating dinner. A subsequent right PhNB was performed a week later, which initially provided significant (>50%) relief. The patient experienced complete relief for approximately eight hours, but when the hiccups returned, he described the subsequent episode as "the worst ever." This significant worsening after the right-sided block may have represented a paradoxical effect or unmasking of contralateral (left-sided) diaphragmatic dysfunction.

Following this episode, bilateral phrenic nerve peripheral nerve stimulation (PNS) (Sprint, SPR Therapeutics, Cleveland, OH, USA) was attempted for 60 days but provided no symptomatic benefit. The patient also trialed a handheld noninvasive vagus nerve stimulator device (Truvaga, Rockaway, NJ, USA), which was similarly ineffective. As a result, the treatment plan was modified to include sequential right- and left-sided stellate ganglion blocks (SGB), but the patient was ultimately lost to follow-up.

Case 4

A 46-year-old African American male presented with chronic hiccups that had persisted for four years without any identifiable precipitating event. The hiccups were constant throughout the day and only ceased when the patient gagged. The episodes were triggered by arm-stretching exercises, overeating, and deep breaths, with symptoms reportedly more concentrated on his right side. Because the hiccups predated his surgical history by several years, they were unlikely to have been caused by prior operative intervention; however, his complex postoperative anatomy may have increased procedural risk and made the PhNB technically more challenging.

The patient had a significant gastrointestinal history, including a small hiatal hernia discovered on endoscopy. Earlier in the year, he had undergone a hiatal hernia repair and Nissen fundoplication, but two days post-procedure, he required an emergency laparotomy for suspected gastric strangulation. This led to a one-month hospitalization and feeding via a G-tube, which was eventually removed. He continued to report dysphagia to both solids and liquids and reflux symptoms, which were managed with omeprazole 40 mg three times a day. The patient actively avoided trigger foods, including carbonated drinks, chocolate, and spicy foods. Previous interventions included a left SGB two years prior, which provided no relief, and medication management with baclofen and chlorpromazine, which had minimal benefit.

The procedure was initially uneventful; however, approximately 20 minutes after leaving the clinic, the patient's girlfriend called to report that he was experiencing a severe hiccup episode and difficulty breathing. Upon return to the office, the patient was ambulatory and hemodynamically stable, with oxygen saturation in the high 90s-100%, a heart rate around 120 bpm, and blood pressure in the 110-120/70-80 range. He was treated for the acute episode with intravenous promethazine 25 mg, intravenous labetalol 10 mg, and nitrous-oxide inhalation therapy, which reduced the intensity and frequency of hiccups. He was monitored for 30 minutes in the clinic and subsequently discharged home once stable.

Although the exact mechanism of this adverse event could not be definitively determined, it was hypothesized to represent a transient vagal or sympathetic response rather than direct phrenic or respiratory compromise, given the absence of desaturation or hemodynamic instability. After a full recovery and following detailed discussion of risks and alternatives, a contralateral (left-sided) PhNB was performed two days later under a modified, lower-risk protocol. Specifically, nerve stimulation was omitted to minimize procedural irritation, and a smaller volume of 3 mL of 1% lidocaine with 4 mg perineural dexamethasone was used. The patient tolerated the procedure well, with partial symptomatic improvement, including delayed onset of triggers and reduced hiccup severity, though he noted his symptoms now felt more "gastric," with increased belching. The patient was offered a phrenic nerve catheter for ongoing management but deferred the procedure.

Case 5

A 50-year-old African American male and professional bodybuilder with a history of hypertension and a defibrillator implant presented with chronic hiccups that had persisted for four months. The hiccups typically occurred postprandially, with vomiting being the only intervention that provided relief. Although the condition had initially been intermittent, it had become constant by the time of presentation, significantly affecting his sleep.

The previous diagnostic workup included imaging studies and a colonoscopy, neither of which revealed an identifiable cause. Previous treatment attempts with medications such as chlorpromazine and muscle relaxants provided minimal to no relief. A trial of a handheld vagus nerve PNS device was also attempted but proved ineffective.

A left PhNB was performed using 3 mL of 0.25% ropivacaine combined with 8 mg of perineural dexamethasone under ultrasound guidance. The higher dexamethasone dose was selected to prolong the duration of the block, given the patient's muscular body habitus and thick cervical musculature, which were expected to increase local anesthetic dispersion and reduce block efficacy. The procedure was completed without complications; however, follow-up the next day revealed a post-procedural exacerbation of symptoms, with worsening hiccups and severe insomnia.

This paradoxical worsening may represent transient diaphragmatic or phrenic nerve irritation following the block and may result from temporary neural disinhibition or contralateral compensatory activation. The patient's girlfriend initially left a voicemail reporting symptom worsening, followed by a subsequent call from the patient requesting to speak with the physician. Despite multiple outreach attempts, the patient did not return for in-person evaluation or additional follow-up.

Case 6

A 59-year-old African American male with a history of right rotator cuff repair, total hip replacement, and left meniscus repair presented to the clinic with chronic hiccups persisting for eight months, accompanied by vomiting for the past three months, resulting in a 20 lb weight loss. The patient reported that eating triggered vomiting episodes, which then led to relentless hiccups and severe pain. Previous treatment attempts with various medications were unsuccessful, though he noted partial relief with gabapentin and acetaminophen.

On examination, the patient appeared exhausted due to constant hiccuping and reported significant fatigue, sleep disturbance, weight loss, and poor appetite. Psychiatric review revealed depressive symptoms, insomnia, and passive suicidal ideations.

**Figure 1:**
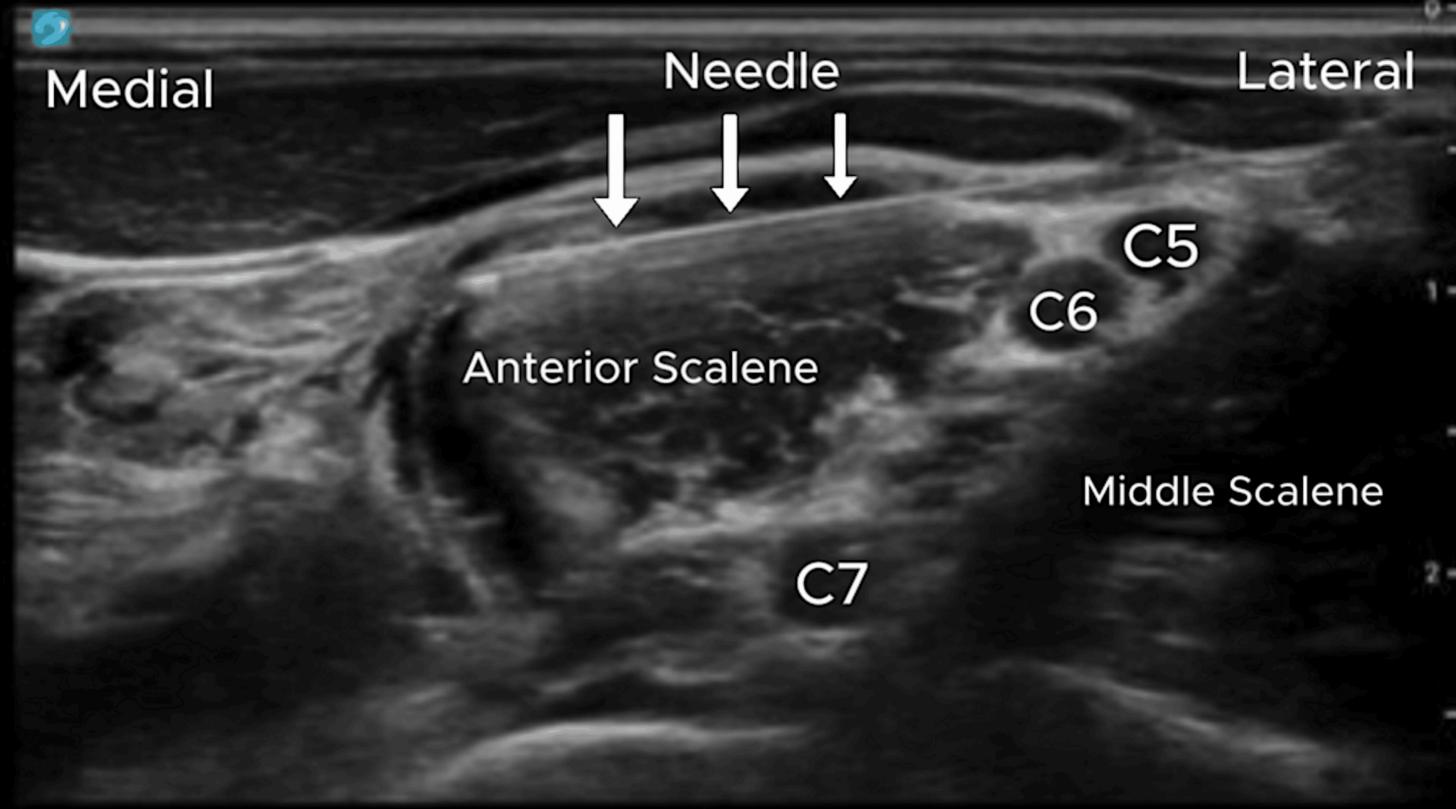
Ultrasound image demonstrating right phrenic nerve block (PhNB). The image labels anatomical landmarks, including the anterior and middle scalene muscles, cervical vertebrae (C5, C6, C7), and needle trajectory (white arrows). The phrenic nerve is typically located anterior to the anterior scalene muscle. This image is original and was obtained from one of the cases included in this study.

A left PhNB was performed using 3 mL of 0.25% ropivacaine and 4 mg dexamethasone under ultrasound and nerve stimulator guidance (Figure 1). The procedure produced an immediate >75% reduction in hiccup severity, and at a five-day follow-up, the patient reported a sustained >50% reduction in both severity and frequency.

During that visit, a right PhNB was performed using the same protocol. The procedure was completed uneventfully and was well tolerated, with no immediate post-procedural complications. The patient was discharged home with routine monitoring instructions and a follow-up scheduled in one week. However, the patient did not return for follow-up evaluation, and no additional long-term outcome data are available.

Case 7

A 27-year-old African male currently employed as a fisherman presented with chronic hiccups persisting for eight months. The patient reported constant hiccups throughout the day that worsened with eating or drinking. While the hiccups would occasionally cease for a few days, the patient was unable to control them independently. He had lost 10 lb over an eight-month period and developed acid reflux. The patient had a known history of hiatal hernia since birth, which his gastroenterologist had determined did not require intervention. Prior diagnostic workup, including endoscopy and colonoscopy, was otherwise unremarkable. Previous treatment attempts with baclofen were unsuccessful due to excessive drowsiness, and chlorpromazine and gabapentin provided no relief.

On initial examination, the patient was experiencing active hiccups. A left PhNB was performed using 4 mL of 0.25% ropivacaine and 4 mg of perineural dexamethasone under ultrasound guidance, resulting in cessation of hiccups originating from the left hemidiaphragm. At follow-up the next day, the patient reported reduced hiccup intensity, primarily localized to the right side, though he also experienced increased regurgitation after meals, likely related to transient left hemidiaphragm relaxation.

Given persistent right-sided symptoms and the patient's request for further relief, a right PhNB was subsequently performed using the same protocol. Recognizing the potential risk of bilateral hemidiaphragmatic paresis and its implications for respiratory function, intra-abdominal pressure generation, and swallowing coordination, the procedure was conducted with careful dose control, ultrasound guidance, and continuous cardiorespiratory monitoring. The patient remained hemodynamically stable and maintained normal oxygen saturation throughout the procedure and observation period.

The block resulted in immediate cessation of hiccups; however, the patient later developed right-sided Horner's syndrome, presumed secondary to sympathetic chain involvement. No respiratory distress or swallowing difficulty was reported at the time of discharge. The side effects had not yet resolved at the time of last contact, and the patient was lost to follow-up thereafter.

## Discussion

Intractable hiccups can stem from various causes, with one of the most frequent being the rapid expansion of the stomach, often triggered by quickly consuming a large meal or drinking carbonated beverages. Physiologically, hiccups arise from erratic stimulation of the reflex arc, which includes afferent pathways involving the phrenic, vagus, and sympathetic nerves (T6-T12) that transmit signals to the central processing regions in the midbrain, particularly the periaqueductal gray (PAG).

The central processing of hiccups remains poorly understood: it may be processed solely by the brainstem or by other portions of the central nervous system (CNS) between the brainstem and the cervical spine [7]. Efferent pathways of the reflex arc involve the phrenic nerve, which innervates the diaphragm, and the accessory nerve, which controls the intercostal muscles. Among the neurotransmitters that are involved in the hiccup reflex arc, both gamma-aminobutyric acid (GABA) and dopamine (D) have been implicated [8]. These pathways lead to sudden contractions, producing the characteristic "hic" sound [9,10]. Due to the large amount of afferent and efferent pathways that are involved in the reflex arc, the exact etiology of intractable hiccups is very difficult to diagnose [11]. Even in cases where the hiccup reflex is centrally mediated, PhNB may provide symptomatic relief by interrupting the final efferent pathway responsible for diaphragmatic contraction, thereby temporarily disrupting the reflex arc and potentially "resetting" abnormal central activity.

In general, intractable hiccups fall into three categories: organic, idiopathic, and psychogenic. Organic causes are due to peripheral nervous system (PNS) disorders derived from either phrenic or vagus nerve stimulation [12]. One common cause of intractable hiccups is CNS lesions, which may result from various etiologies, including vascular diseases, tumors, inflammation, and seizures [13-15]. Another potential cause of hiccup reflex arc dysfunction is impairment of the PNS pathway. Conditions such as mediastinal diseases, myocardial ischemia, gastroesophageal reflux disease (GERD), and esophageal tumors have been implicated in disrupted PNS signaling [16-18].

Idiopathic causes of phrenic nerve dysfunction include exposure to anesthetic and chemotherapeutic agents, anti-Parkinsonian medications, and cardiovascular catheter ablations [19-22]. Idiopathic and psychogenic etiologies are more elusive and do not have consistent presenting signs, so various treatments have been suggested to treat them. Pharmacological treatments include but are not limited to baclofen, chlorpromazine, metoclopramide, and haloperidol [10,23]. Non-pharmacological and non-invasive first-line treatments include, but are not limited to, digital rectal stimulation, carotid sinus massage, and irritation of the uvula [12].

However, when these are ineffective, a PhNB has been suggested as a possible therapeutic interventional treatment. The proposed mechanism of action of the PhNB is to induce neural pathway interruption from the anterior branch of spinal root C4 when it is traveling caudally through the anterior scalene and omohyoid muscles [24]. This can be done via an ultrasound-guided local anesthetic injection and/or electrical nerve stimulation [9,10]. Key aspects of the procedure include supine patient positioning, with the head turned away from the side being blocked. Ultrasound guidance is typically used to locate the nerve and surrounding structures, and an anesthetic is carefully injected around the nerve (Video 1). Bilateral phrenic nerve procedures should be approached with caution, and transient diaphragmatic weakness on both sides may increase the risk of respiratory compromise. Therefore, careful patient selection, pulmonary function testing, and staged procedures are essential to minimize these risks.

**Video 1:** Ultrasound recording of the right lateral neck demonstrating anatomical landmarks for a phrenic nerve block. The phrenic nerve is visualized along the anterior surface of the anterior scalene muscle, deep to the sternocleidomastoid. The carotid artery (CA) is seen medially, with the middle scalene positioned laterally. This view guides safe and accurate needle placement for targeted anesthetic delivery. This video is original and was recorded as part of the present case series.

Another therapeutic modality that has been emerging as an intervention for intractable hiccups includes an SGB. The stellate ganglion, formed by the fusion of the inferior cervical and first thoracic sympathetic ganglia, plays a crucial role in sympathetic innervation of the head, neck, and upper extremities. By administering a local anesthetic to this ganglion, SGB effectively inhibits sympathetic nerve activity, which may disrupt the hiccup reflex arc and alleviate symptoms [25].

Several case reports have documented the successful use of SGB in treating persistent hiccups. For instance, a case report reported a patient with intractable hiccups following an epidural steroid injection who got complete resolution of his hiccups with an SGB [26]. Similarly, another case series demonstrated the efficacy of SGB in resolving postoperative hiccups unresponsive to standard medical therapies [27]. Although its precise mechanism of action is unknown, given its minimally invasive nature and potential benefits, SGB represents a promising option for patients suffering from refractory hiccups.

PNS devices have also been described as helpful for post-surgical and chronic pain; additionally, PNS has shown promise as a tool to "reset" the hiccup reflex arc and provide therapeutic release for patients with intractable hiccups [2]. As seen in our third patient, the implantation of a SPRINT PNS device had no lasting benefit, which underscores the view that this is still an experimental device and cannot be generalized to the broader patient population without further studies of the device's efficacy for reducing intractable hiccups. Our third patient also had a Truvaga vagal nerve stimulation device scheduled for implantation. It has been hypothesized that the vagus nerve is part of the reflex arc underpinning hiccups; this stimulation device disrupts the reflex arc using nerve stimulation [28]. However, there have been reports of vagal PNS devices also causing and exacerbating intractable hiccups, highlighting the ambiguity of PNS devices as a therapeutic option for this condition [29].

When PhNBs are ineffective, spontaneous recurrence of intractable hiccups can occur. A subsequent repeat PhNB has been proposed as an effective treatment to reduce the severity of the recurrent hiccups [23]. However, as seen in the third case, a repeat nerve block did not reduce the severity or the frequency of hiccups, and recurrence occurred less than 24 hours after PhNB. Even further, our sixth case was willing to undergo bilateral PhNBs in order to prevent recurrence of their symptoms, although their outcomes have not been comprehensively assessed due to the recency of their PhNB procedures.

Additionally, since PhNB does not have wide acceptance of practice yet, data on the minimum effective dosage of anesthetics are limited. In our case series, we used either ropivacaine or lidocaine, which is in line with prior practice seen in the literature, although some case reports have also described using bupivacaine [2,28]. Further studies are warranted to define an appropriate dose and duration of therapy (i.e., infusion using a catheter), as well as complications associated with various dosages, as currently used methods could potentially be ineffective or inappropriate for patients with intractable hiccups.

In each of our patients, we were careful to note the important anatomical structures surrounding the nerve. Ultrasound guidance was used for every injection, with proper identification of the anterior and middle scalene muscles, brachial plexus nerves and phrenic nerves during each injection. If local anesthetic flowed into the interscalene space, it could cause an unintended brachial plexus block, leading to unexpected sensory and motor loss in the upper limbs. Other unintended adverse effects could include symptoms of tachycardia or hoarseness from recurrent laryngeal nerve and vagus nerve injury [3].

Nonetheless, another unintended adverse effect of PhNB is Horner's syndrome, as seen in our seventh case, due to a sympathetic chain blockade. Horner's syndrome classically presents with ptosis, miosis, and anhidrosis, is typically self-resolving, and usually manifests in a unilateral distribution; in our patient, it was identified on the ipsilateral side of the PhNB [29]. The cervical sympathetic chain, which is affected in Horner's syndrome, lies deep to the prevertebral fascia. During a PhNB, the anesthetic injected above the prevertebral fascia can penetrate through pores and disperse into deeper areas of the neck, including the cervical sympathetic chain, causing inadvertent Horner's syndrome [29].

Notably, our fourth patient also had an adverse effect from the procedure, which resulted in a severe hiccup episode; the patient was stable and in no respiratory distress but was administered IV medications, which reduced the frequency and intensity of his hiccups. Respiratory depression and resultant pulmonary function depression have been documented as additional adverse effects in PhNBs, showing declines in forced expiratory volume in 1 second (FEV1), forced vital capacity (FVC), and peak expiratory flow (PEF) [2]. Our outpatient facility was not equipped with the ability to assess pulmonary function using FEV1/FVC/PEF; however, close follow-up is warranted to make sure that patients do not develop any of these unwanted side effects due to PhNB.

In our case series, five of the seven patients were of African descent (one African, one Caribbean, and three African American), and all were male, with five reporting regular bodybuilding or weight training activity. While this demographic clustering is observational, no evidence currently supports a specific ethnic predisposition to intractable hiccups, and this finding should be interpreted cautiously. The association between muscular body habitus and persistent hiccups remains speculative; however, diaphragmatic hypertrophy in athletic individuals may theoretically increase diaphragmatic excitability or mechanical sensitivity, potentially contributing to symptom persistence. A prior case report described intractable hiccups in a competitive bodybuilder using anabolic steroids, attributed to central efferent limb activation involving the medullary reticular formation and respiratory centers [30].

Limitations

This study has several limitations that should be acknowledged. First, the small sample size of seven patients limits the generalizability of the findings and precludes any meaningful statistical analysis. The retrospective and descriptive design introduces inherent biases, including variability in follow-up duration, incomplete documentation, and heterogeneity in patient characteristics such as duration, etiology, and severity of hiccups. The absence of standardized procedural protocols and objective outcome measures, such as validated scales for hiccup frequency, intensity, or quality of life, further restricts the ability to quantify treatment response consistently across patients.

Another important limitation is the high proportion of incomplete or transient responses observed, which highlights the variable and unpredictable efficacy of PhNB in real-world clinical practice. The study was also limited by operator dependency and potential selection bias, as all procedures were performed by a single physician without uniform technique or dosing protocols. Documentation of adverse events and post-procedural monitoring data was inconsistent, reflecting the constraints of retrospective record review. In addition, long-term follow-up data were unavailable for several patients who were lost to follow-up, preventing a comprehensive assessment of symptom durability and delayed complications.

Finally, the study's findings differ from prior case reports describing higher PhNB success rates. This discrepancy may reflect differences in patient selection, underlying pathophysiology, or procedural technique, rather than a contradiction of existing evidence. Future prospective, controlled studies with standardized procedural parameters, patient selection criteria, and objective outcome metrics are needed to better define the clinical role and safety profile of PhNB in managing intractable hiccups.

## Conclusions

This case series highlights the variability in responses to PhNB for intractable hiccups. While a subset of patients experienced meaningful symptomatic improvement, others demonstrated minimal benefit or transient worsening, underscoring that PhNB may not be universally effective. Accordingly, PhNB should be considered an exploratory or adjunctive intervention rather than a definitive therapy, ideally reserved for carefully selected, refractory cases where other modalities have failed.

Further investigation into alternative interventional strategies, such as vagus nerve stimulation or SGB, and the development of standardized procedural and patient selection protocols will be essential to better define the safety profile, mechanism, and clinical utility of PhNB in this population. Ultimately, a cautious, individualized approach guided by clinical judgment and evolving evidence remains paramount.
